# Resection of a rapidly growing chest wall cyst: a case report

**DOI:** 10.1186/s44215-023-00121-7

**Published:** 2023-11-16

**Authors:** Shumpei Kato, Takashi Sakai, Megumi Kusano, Satoshi Koezuka, Hajime Otsuka, Yoko Azuma, Yasuko Kurose, Naobumi Tochigi, Akira Iyoda

**Affiliations:** 1 Division of Chest Surgery, Department of Surgery, Toho University School of Medicine, 6-11-1, Omorinishi, Ota, Tokyo, 143-8541 Japan; 2 Department of Surgical Pathology, Toho University School of Medicine, Tokyo, Japan

**Keywords:** Chest wall cyst, Angiogenesis, Seroma

## Abstract

**Background:**

When cystic lesions are detected in the chest wall, postoperative seroma and abscesses can be considered in the differential diagnoses. Herein, we present a case of a large chest wall cyst with rapid growth 5 years after thoracic surgery.

**Case presentation:**

A male patient in his 60s was admitted to our hospital complaining of a rapidly enlarging chest wall swelling. He underwent a pleural biopsy for pleural thickening and was diagnosed with pleurisy 5 years ago. Computed tomography revealed a cystic lesion with a capsule measuring 14 cm × 10 cm, and contrast-enhanced magnetic resonance imaging revealed a heterogeneous enhancement effect inside the lesion. Surgical resection was performed for definitive diagnosis and therapeutic purposes. Intraoperatively, a cystic lesion with a thick capsule located outside the thorax was seen. The lesion was resected completely with no remaining adherence to the surrounding tissues and muscles. Histopathological examination revealed that the cyst wall was a non-epithelial fibrous connective tissue with inflammatory cell infiltration, and the contents were viscous liquid and fibrin, suggestive of a chest wall cyst. The cyst wall had abundant CD34-positive vascular endothelium, suggestive of rapid enlargement due to the influx of exudate associated with angiogenesis. No recurrence was observed 1 year postoperatively.

**Conclusions:**

In addition to bacteriology, pathological examination, including immunohistological examination, is useful for the differential diagnosis of chest wall cystic lesions.

## Introduction

There are several reports of infectious inflammatory lesions and abscesses in chest wall cystic lesions, such as bacterial infection caused by trauma and cold abscesses caused by tuberculosis [1, 2]. Postoperative seroma has also been frequently reported. Most postoperative seromas improve with follow-up or puncture drainage alone; however, cases of chronic, encapsulated seromas covered with a thick capsule and those requiring surgical resection have also been reported [3, 4]. In the present case, the encapsulated seroma-like lesion expanded rapidly 5 years after thoracic surgery. We diagnosed the cystic lesion as a noninfectious chest wall cyst via bacteriological, histopathological, and immunological investigations.

## Case presentation

**Fig. 1:**
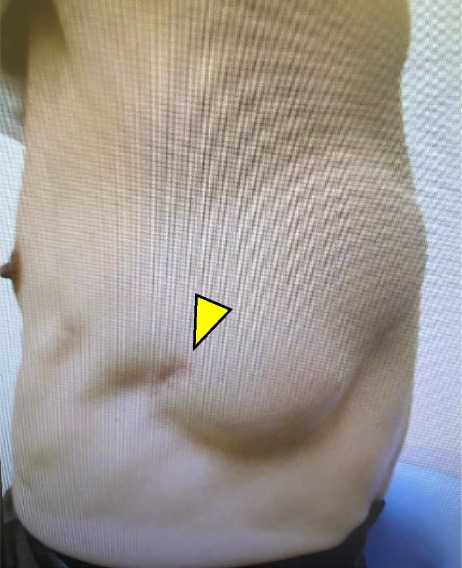
Left-back swelling of the patient. He underwent a video-assisted pleural biopsy with a single port 5 years ago (yellow arrow: port site of the past surgery)

**Fig. 2:**
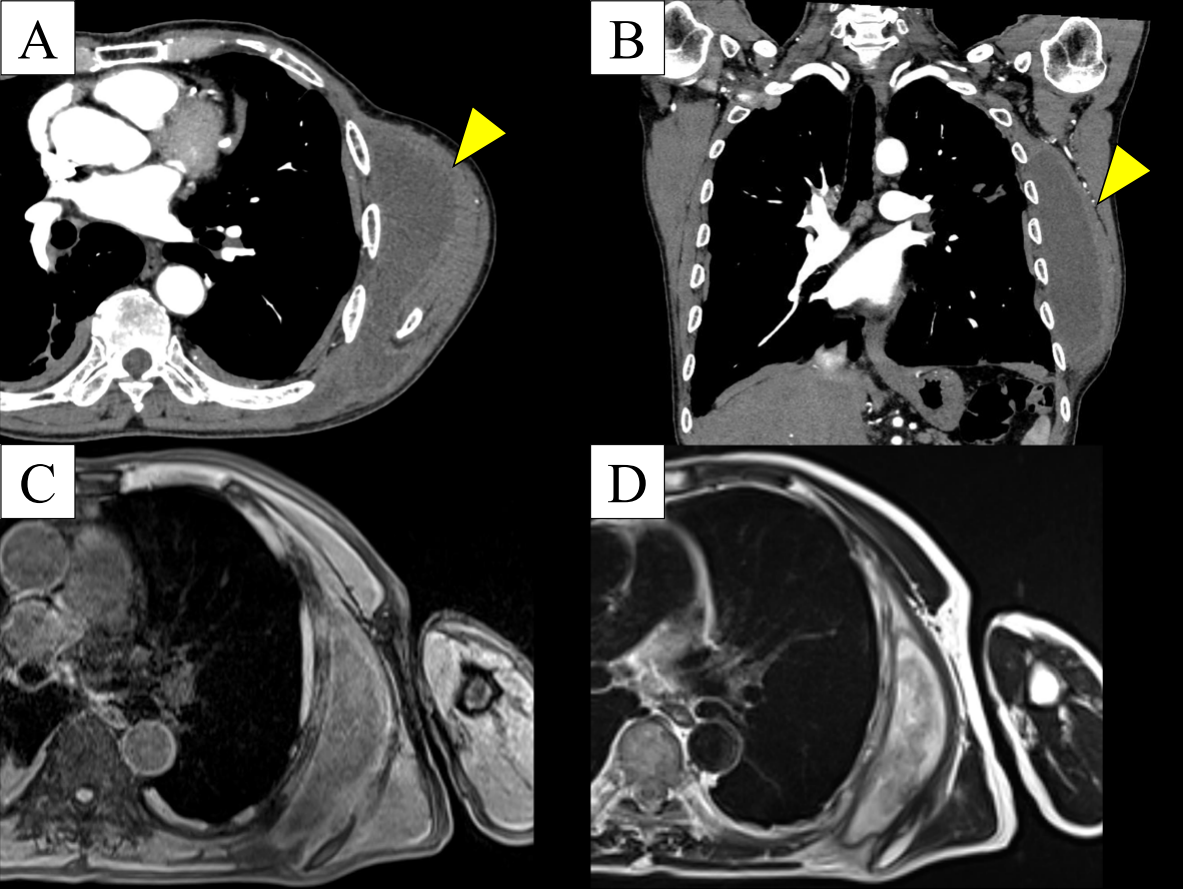
Enhanced computed tomography scan showed a capsulized cystic lesion and substantial ingredients within the lesion (**A**, **B**). Contrast-enhanced magnetic resonance imaging revealed a lesion with a viscous liquid (**C** T1W2, **D** T2W1)

**Fig. 3:**
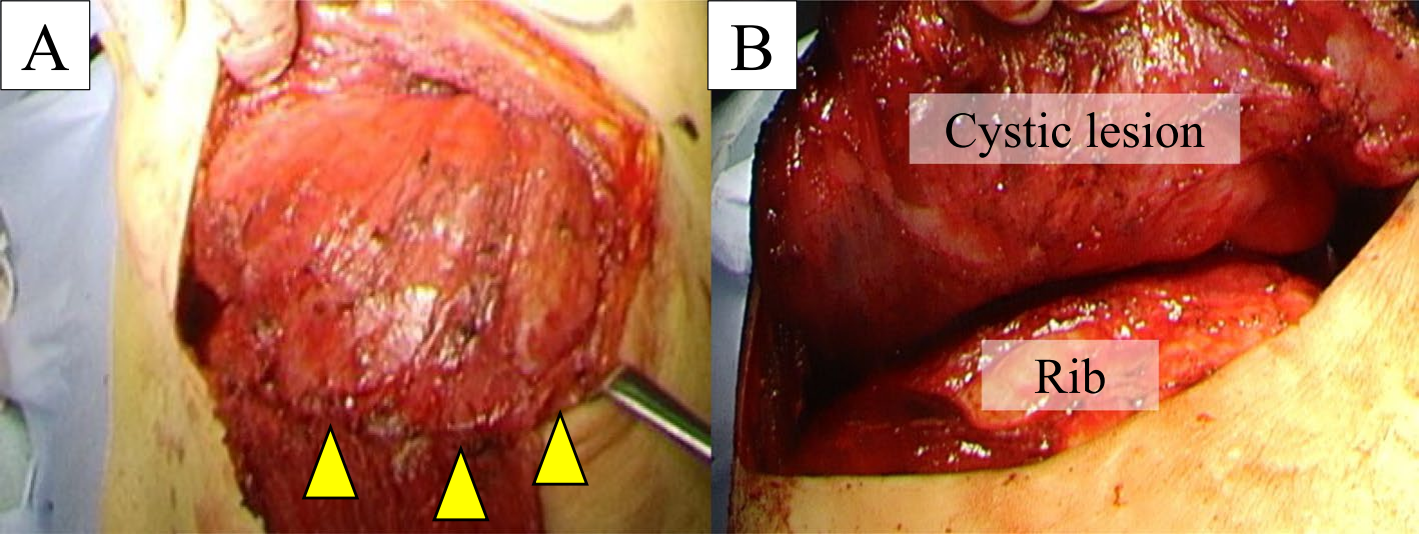
Intraoperative findings. The lesion was an extra-thoracic cyst that was entirely encapsulated (**A**). Adhesions were observed with surrounding tissues and muscles; however, it was not adherent to the thoracic cavity (**B**)

**Fig. 4:**
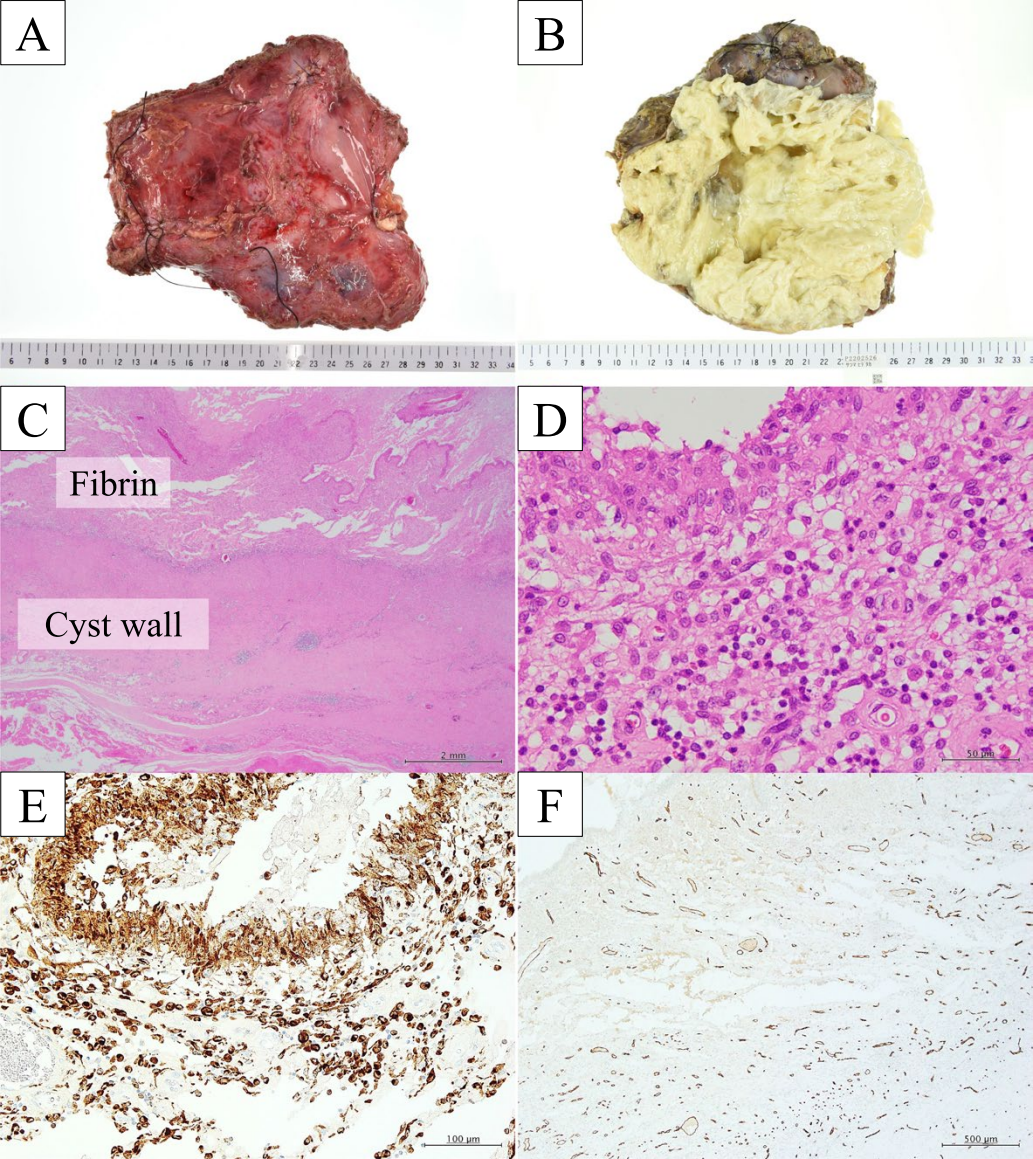
Histopathological findings. The lesion was capsulized with a thick tissue of about 5 mm (**A**) and contained yellowish fibrin and viscous liquid (**B**). The cyst wall was formed by fibrous connective tissue (**C**) and infiltration of lymphocytes and CD68-positive histiocytes (**D** and **E**). The cyst wall had abundant CD34-positive vascular endothelium, suggesting significant angiogenesis (**F**)

A male patient in his 60s was admitted to our hospital with a chief complaint of left back swelling since 1 year (Fig. 1). He underwent a video-assisted pleural biopsy with a single port 5 years prior for left pleural thickening, which was suspected to be malignant pleural mesothelioma. The histopathological diagnosis was chronic pleural inflammation, and bacteria were not identified. Wound healing was delayed postoperatively; however, it healed completely within 2 months. The patient was in good general condition and had untreated diabetes with an HbA1c level of 8.0%. The lesion was elastic and soft, and there were no findings suggestive of infection, including tenderness or fever. The lesion was 14 × 10 cm in diameter and increased by 5 cm in 3 months. Blood tests showed that the white blood cell count was within the normal range, and a slight elevation of c-reactive protein level was noted (1.3 mg/dl). An enhanced computed tomography (CT) scan showed a capsulized cystic lesion with substantial ingredients within it (Fig. 2A and B). Contrast-enhanced magnetic resonance imaging revealed a lesion containing viscous liquid (Fig. 2C and D). Cytological examination via lesion puncture was attempted; however, the contents could not be aspirated. Thus, surgical resection was performed for definite diagnosis and treatment. Intraoperative findings showed that the lesion was an extra-thoracic cyst with complete encapsulation (Fig. 3A). Adhesions were observed with the surrounding tissues and muscles; however, they were not adherent to the thoracic cavity (Fig. 3B). The patient was discharged on postoperative day 7 without any complications and 1 year passed without recurrence. Pathological examination revealed that the lesion was capsulized with a thickness of approximately 5 mm and contained yellowish fibrin and viscous liquid (Fig. 4A and B). The cyst wall was formed by fibrous connective tissue (Fig. 4C) and infiltration of lymphocytes and CD68-positive histiocytes (Fig. 4D and E). Caseous necrosis, which is shown in thoracic tuberculosis, was not detected. Immunohistological examination using AE1/AE3 did not reveal epithelium on the cyst wall. The CD34-positive vascular endothelium was abundant in the cyst wall, suggesting significant angiogenesis (Fig. 4F). These results suggest that cysts may have been formed by fibrous connective tissue accompanied by histiocyte-dominated inflammatory cell infiltration and rapidly increased exudate production associated with angiogenesis.

## Discussion

Infectious inflammatory lesions and abscesses, such as bacterial infections caused by trauma and cold abscesses caused by tuberculosis, are the most common non-neoplastic cystic lesions of the chest wall [1, 2]. In acute abscess formation due to normal bacterial infection, fever and increased inflammatory reaction are observed clinically, and inflammatory cell infiltration mainly comprising neutrophils is observed on pathological examination [1]. Chest tuberculosis is sometimes seen as cystic enhancement effects on CT images and has clinical findings that differ from bacterial infection, such as the absence of fever and pain and no increase in inflammatory reaction [5]. Additionally, a review of 80 cases of thoracic tuberculosis confirmed caseous necrosis in all pathological examinations [2].

As a non-infectious lesion, encapsulated seroma has been reported as a condition in which a pseudocyst formed by non-epithelial connective tissue contains exudates mainly comprising lymphocytes [3]. Seroma usually refers to the accumulation of lymphatic fluid and exudate in the dead space created by trauma or surgery [6]. As for the mechanism of development, it is thought that the primary healing delay in the dead space precedes the secondary healing delay, and the dead space is covered with granulation tissue to form a pseudocyst wherein serous fluid accumulates [6]. When seromas become chronic, they rarely become encapsulated seromas surrounded by thick septums [3]. Such conditions have been reported after many surgeries [7–10]. However, the pathogenesis of encapsulated seroma has not been elucidated, and the distinction between ordinary seroma and encapsulated seroma has not been defined [3]. The treatment strategy differs between encapsulated seroma and ordinary seroma in that surgery is selected in many cases when the seroma is thickly encapsulated.

In the present case, the cystic wall comprised non-epithelial fibrous connective tissue containing inflammatory exudates, such as lymphocytes and histiocytes, suggesting an encapsulated seroma. Inflammatory lesions were suspected because no epithelial cells were detected, angiogenesis was remarkable, and they contained exudate containing many lymphocyte-dominant inflammatory cells. However, bacteria were not detected in pathological and culture tests, and the lesion was diagnosed as a non-infectious chest wall cyst. Such findings have never been reported before; we have searched for similar patients using keywords such as “cyst and chest wall,” “seroma and chest wall,” and “abscess and chest wall” via PubMed. We suggest that the formation of the lesion resulted in delayed wound healing during the initial surgery. Delayed wound healing at the port site may have resulted in dead space and seroma. The presence of diabetes may also increase the risk of developing seroma [4]. Although the lesion was nonspecific, it grew at a rapid rate a long time after surgery; thereon, the lesions became chronic during the course of the disease, and an inflammatory reaction was induced by some trigger, resulting in a rapid increase in size due to the influx of exudate associated with angiogenesis.

## Data Availability

The case report and patient consent form are available on reasonable request from the corresponding author.

## References

[1] Yamaoka Y, Yamamura J, Masuda N, Yasojima H, Mizutani M, Nakamori S, et al. Primary chest wall abscess mimicking a breast tumor that occurred after blunt chest trauma: a case report. Case Rep Med. 2014; 10.1155/2014/620876. 10.1155/2014/620876 PMC3934081 24660001

[2] Kim YT, Han KN, Kang CH, Sung SW, Kim JH. Complete resection is mandatory for tubercular cold abscess of the chest wall. Ann Thorac Surg. 2008;85:273–7. 18154822 10.1016/j.athoracsur.2007.08.046

[3] Kazzam ME. Ng P: Postoperative seroma management. In: Island AT, editor. *StatPearls*. (FL): StatPearls Publishing; 2022. 36256748

[4] Unger J, Rutkowski R, Kohlmann T, Paepke S, Zygmunt M, Ohlinger R. Potential risk factors influencing the formation of postoperative seroma after breast surgery - a prospective study. Anticancer Res. 2021;41:859–67. 33517291 10.21873/anticanres.14838

[5] Sonhaye L, Amadou A, Gnandi-Piou F, Assih K, Tchaou M, Kolou B, et al. Tuberculous abscess of the chest wall simulate pyogenic abscess. Case Rep Radiol. 2015; 10.1155/2015/195412. 10.1155/2015/195412 PMC4651642 26618019

[6] Kuroi K, Shimozuma K, Taguchi T, Imai H, Yamashiro H, Ohsumi S, et al. Pathophysiology of seroma in breast cancer. Breast Cancer. 2005;12:288–93. 16286909 10.2325/jbcs.12.288

[7] Goldman A, Wollina U, França K, Tchernev G, Lotti T. Chronic encapsulated seroma persisting for three years after abdominoplasty and a successful surgical solution. Open Access Maced J Med Sci. 2018;6:82–4. 29483991 10.3889/oamjms.2018.051 PMC5816325

[8] Dujmović A, Jurišić N, Orehovec SS, Vrbanović Mijatović V, Mijatović D. Pseudocyst formation after abdominoplasty: a case report. Acta Clin Croat. 2021;60:548–51. 10.20471/acc.2021.60.03.28 PMC8907945 35282498

[9] Tanaka K, Oura S, Yasuda K, Makimoto S. Abrupt aggravation of encapsulated seroma after breast reconstruction with extended latissimus dorsi muscle flap. Case Rep Oncol. 2021;14:290–5. 33776719 10.1159/000513491 PMC7983582

[10] Fosheim K, Bojesen S, Troestrup H, Laenkholm A. Capsulectomy can successfully treat chronic encapsulated breast seroma: a case report. Cureus. 14:e21677. 10.7759/cureus.21677. 10.7759/cureus.21677 PMC8882229 35237476

